# Characterization of *cp3 *reveals a new *bri1 *allele, *bri1-120*, and the importance of the LRR domain of BRI1 mediating BR signaling

**DOI:** 10.1186/1471-2229-11-8

**Published:** 2011-01-11

**Authors:** Yun Shang, Myeong Min Lee, Jianming Li, Kyoung Hee Nam

**Affiliations:** 1 Division of Biological Science, Sookmyung Women's University, Seoul, Korea; 2 College of Life Science and Biotechnology, Yonsei University, Seoul, Korea; 3 Department of Molecular, Cellular, and Developmental Biology, University of Michigan, Ann Arbor, MI, USA

## Abstract

**Background:**

Since the identification of BRI1 (BRASSINOSTEROID-INSENSITIVE1), a brassinosteroids (BRs) receptor, most of the critical roles of BR in plant development have been assessed using various *bri1 *mutant alleles. The characterization of individual *bri1 *mutants has shown that both the extracellular and cytoplasmic domains of BRI1 are important to its proper functioning. Particularly, in the extracellular domain, regions near the 70-amino acid island are known to be critical to BR binding. In comparison, the exact function of the leucine rich-repeats (LRR) region located before the 70-amino acid island domain in the extracellular cellular portion of BRI1 has not yet been described, due to a lack of specific mutant alleles.

**Results:**

Among the mutants showing altered growth patterns compared to wild type, we further characterized *cp3*, which displayed defective growth and reduced BR sensitivity. We sequenced the genomic DNA spanning *BRI1 *in the *cp3 *and found that *cp3 *has a point mutation in the region encoding the 13^th ^LRR of BRI1, resulting in a change from serine to phenylalanine (S399F). We renamed it *bri1-120*. We also showed that overexpression of the wild type BRI1 protein rescued the phenotype of *bri1-120*. Using a GFP-tagged *bri1-120 *construct, we detected the *bri1-120 *protein in the plasma membrane, and showed that the phenotypic defects in the rosette leaves of *bri1-301*, a kinase-inactive weak allele of *BRI1*, can be restored by the overexpression of the *bri1-120 *proteins in *bri1-301*. We also produced *bri1-301 *mutants that were wild type in appearance by performing a genetic cross between *bri1-301 *and *bri1-120 *plants.

**Conclusions:**

We identified a new *bri1 *allele, *bri1-120*, whose mutation site has not yet been found or characterized. Our results indicated that the extracellular LRR regions before the 70-amino acid island domain of BRI1 are important for the appropriate cellular functioning of BRI1. Also, we confirmed that a successful interallelic complementation occurs between the extracellular domain mutant allele and the cytoplasmic kinase-inactive mutant allele of *BRI1 in vivo.*

## Background

Numerous plant developmental processes, such as germination, cell elongation, photomorphogenic responses, and male fertility are regulated by the plant-specific steroidal hormones, brassinosteroids (BR). BR-biosynthetic or BR-perceiving mutants have exhibited defective growth patterns in various tissues that persist throughout their entire life span, indicating the critical role of BR in plant development [1,2]. Although studies researching the BR signaling process began much more recently than any of the other plant hormones, the identification of BRASSINOSTEROID-INSENSITIVE1 (BRI1), a receptor of BR [3], and several other important components involved in BR signaling have provided much insight into many important components in plant development [4]. Plasma membrane-localized BRI1 and its co-receptor BRI1-ASSOCIATED KINASE1 (BAK1) are receptor-like serine/threonine kinases containing leucine-rich repeats (LRR-RLKs) [5,6]. N-terminal LRRs are found in the extracellular portion of the plasma membrane. BRI1 constitutively forms a homodimer in the plasma membrane. In the absence of BR, the activity of the BRI1 homodimer is inhibited by the BRI1 kinase inhibitor 1 (BKI1) by binding BKI1 to the C-terminal portion of BRI1. In the presence of BR, BKI1 is released by the direct binding of BR to the 70-amino acid island region in the extracellular domain of BRI1 [7]. Then, BRI1 recruits BAK1, forming heterodimerized-receptor complexes in the plasma membrane [8,9], leading to the activation of the BES1 and BZR1 transcription factors that regulate the expression of the BR-associated genes [2,10,11].

BRI1 is considered to be a master regulator that plays a critical role in the direct binding of BR and subsequent BR signaling processes [12], while BAK1 has been found to be a partner not only for BRI1 but also for other LRR-RLKs, such as FLS2 and EFRs, which are involved in the plant innate immunity responses [13,14]. To date, genetic screening looking for BR-insensitive mutants has resulted in the identification of only two genes, *BRI1 *and *BIN2 *[3,15]. Since the first report of BRI1 in 1997 [3], more than 30 different mutant alleles have been identified in several different Arabidopsis ecotypes, including Col-0, Ws-2, and En-2 during last two decades. Large numbers of mutant alleles that have mutations in various positions of a specific gene provide information regarding how that gene acts, because the mutation sites themselves are indicators of their importance to the functioning of the gene. In that sense, studying multiple mutant alleles of *BRI1 *will be likely to reveal important information regarding its function. Detailed analyses of the characteristics of each mutation have shown that both the extracellular and cytoplasmic domains of BRI1 are required for full BRI1 functioning, because the mutation sites of all of the *bri1 *mutant alleles are dispersed in both an extracellular domain and a cytoplasmic kinase domain [4,16].

The extracellular domain of BRI1 consists of LRRs and a 70-amino acid island containing unique sequences that show little homology to any other protein. Since BRI1 was discovered, it has been considered to have 25 LRRs with a 70-amino acid island flanking the 21st and 22nd LRR. However, Vert et al, (2005) [4] suggested that BRI1 contains 24 LRRs, postulating that the 21st LRR is actually an atypical formation. It appears evident that the region near the 70-amino acid island allows for the extracellular binding of BR. It is interesting to note that most of the mutation sites in the extracellular domain of BRI1 are clustered in the 70-amino acid island domain and in the 4 LRRs situated before the transmembrane domain. There are very few examples of mutant alleles containing defects in the LRR regions that occur before the 70-amino acid island. This may be partially because the mutations in these LRR regions of BRI1 were neglected due to the lack of any discernible phenotypic alterations. Or, at the opposite extreme, they may lethally affect plant development, resulting in no viable mutants for further analyses. Here, we report a new mutant allele of BRI1, *bri1-120*. A point mutation in the region encoding the 13^th ^LRR of BRI1 in *bri1-120 *caused the defective growth and reduced BR sensitivity of the plant. Using this mutant allele, we demonstrated successful interallelic complementation using a kinase-inactive mutant allele, *bri1-301 *and performed a detailed analysis of BR sensitivity.

## Results

### Phenotypic analyses of the weak *bri1*-looking semi-dwarf mutant, *cp3*

**Figure 1:**
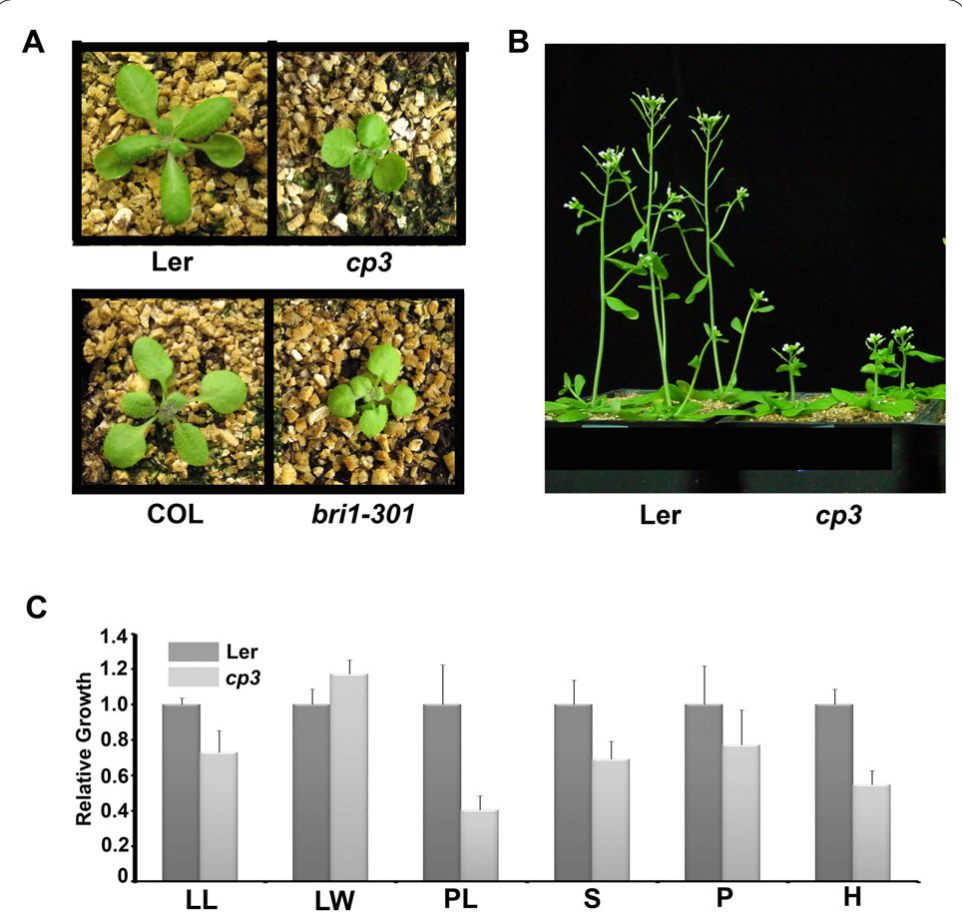
**Phenotypic analysis of *cp3 *mutant compared with weak *bri1 *allele, *bri1-301***. A. 3-week-old soil grown plants of *cp3 *and *bri1-301 *were shown with corresponding wild type plants, Landsberg (Ler) and Columbia (Col), respectively. B. Phenotype of *cp3 *and Ler grown for 5 weeks. C. Quantitative determination of growth in *cp3 *and Ler. Leaf length (LL), leaf width (LW), and petiole length (PL) of the 5-week-old plants were measured Silique length (S), peduncle length (P), and height of individual plants (H) were measured from the 7-week old plants (n = 60, except height (n = 25). Growth is represented as a relative value compared to that of Ler. Experiments were repeated twice. Error bars denote standard errors.

**Figure 2:**
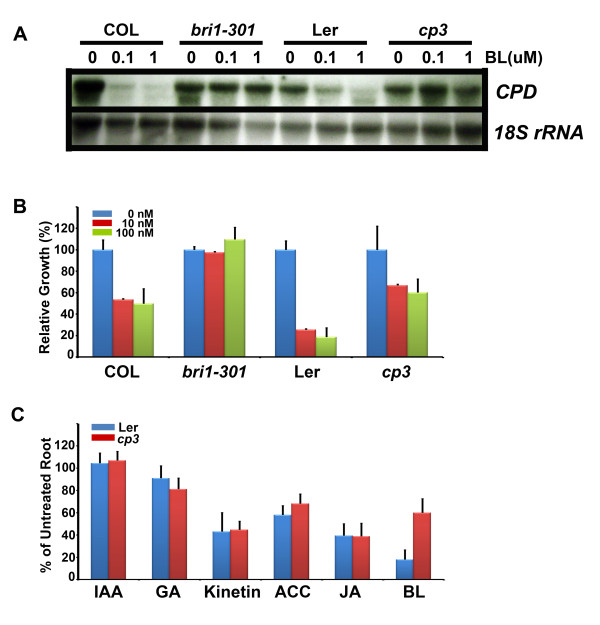
**BR sensitivity of *cp3***. A. Transcriptional inhibition of CPD expression in response to BL was determined in *cp3*, Ler, *bri1-301*, and Col. B. Root growth was measured from the indicated plants grown vertically in 1/2 MS media containing BL. Root length is represented as the relative growth compared to that of the mock-treated sample. C. Root growth was measured from Ler and *cp3 *plants grown vertically in 1/2 MS media containing the various plant hormones indicated. Root length of the plants treated with hormones is represented as a percentage of the root length of the plants grown on the medium without hormone treatment. Experiments were repeated three times. Error bars denote standard errors.

To find natural mutants that show altered growth patterns compared to their corresponding wild type plants, we searched for and obtained mutant seed stocks from the Arabidopsis Biological Resource Center (ABRC). We grew several putative seeds and selected the *cp3 *mutant (seed stock No. CS48) for further analysis, because compared to the corresponding wild type plant Landsberg (Ler), the phenotypic features of the mutant, including the downward curling, dark-green compact rosette leaves, and reduced growth gave the appearance of a weak *bri1 *mutant, *bri1-301 *(Figure 1A and 1B). The *cp3 *mutant exhibited reduced growth in all aspects except leaf width (Figure 1C), resulting in a semi-dwarf stature with round and compact rosette leaves. To determine the BR sensitivity of the *cp3 *mutant, we applied 1 μM of brassinolide (BL), the most bioactive BR, to the mutant plants on the region where the plants were exhibiting growth. Compared with the wild type Ler, which showed elongated petioles and leaves and faded green colored leaves upon overnight BR exposure, the leaves and petioles of the mutant plants were much less elongated and displayed still green-colored leaves, indicating reduced sensitivity to BR (Additional file 1). To confirm this, we analyzed the transcriptional inhibition of the *CPD *expression pattern in *cp3 *mutants with and without exogenous BL treatment, using the known weak *bri1 *mutant, *bri1-301*, as a control (Figure 2A). As observed with *bri1-301*, the *cp3 *mutant contained higher levels of *CPD *transcripts compared to the wild type Ler in the presence of BL, indicating that the *cp3 *mutant possesses reduced-BL sensitivity. We also performed a root growth inhibition assay using the plants grown on the media containing BL (Figure 2B). Both the Columbia (Col-0) and Ler wild type plants showed more than 50% reductions in root growth in the presence of 10 nM BL. In contrast, the *cp3 *mutants showed 30 to 40% reductions in root growth after treatment with the same concentration of BL. In comparison, *bri1-301 *displayed almost no sensitivity to BL in terms of root inhibition. These results indicate that the BL sensitivity of *cp3 *is reduced compared to the wild type, although the degree of reduction is less than that of *bri1-301*. We further assessed the response of *cp3 *to other plant hormones. Similar degrees of root growth inhibition were observed in Ler and *cp3 *when the plants were treated with a variety of hormones with the exception of BL (Figure 2C), indicating that the *cp3 *mutant specifically has a reduced sensitivity to BL, but not to any other plant hormones.

### Identification of the weak *bri1 *mutant allele, *bri1-120*

**Figure 3:**
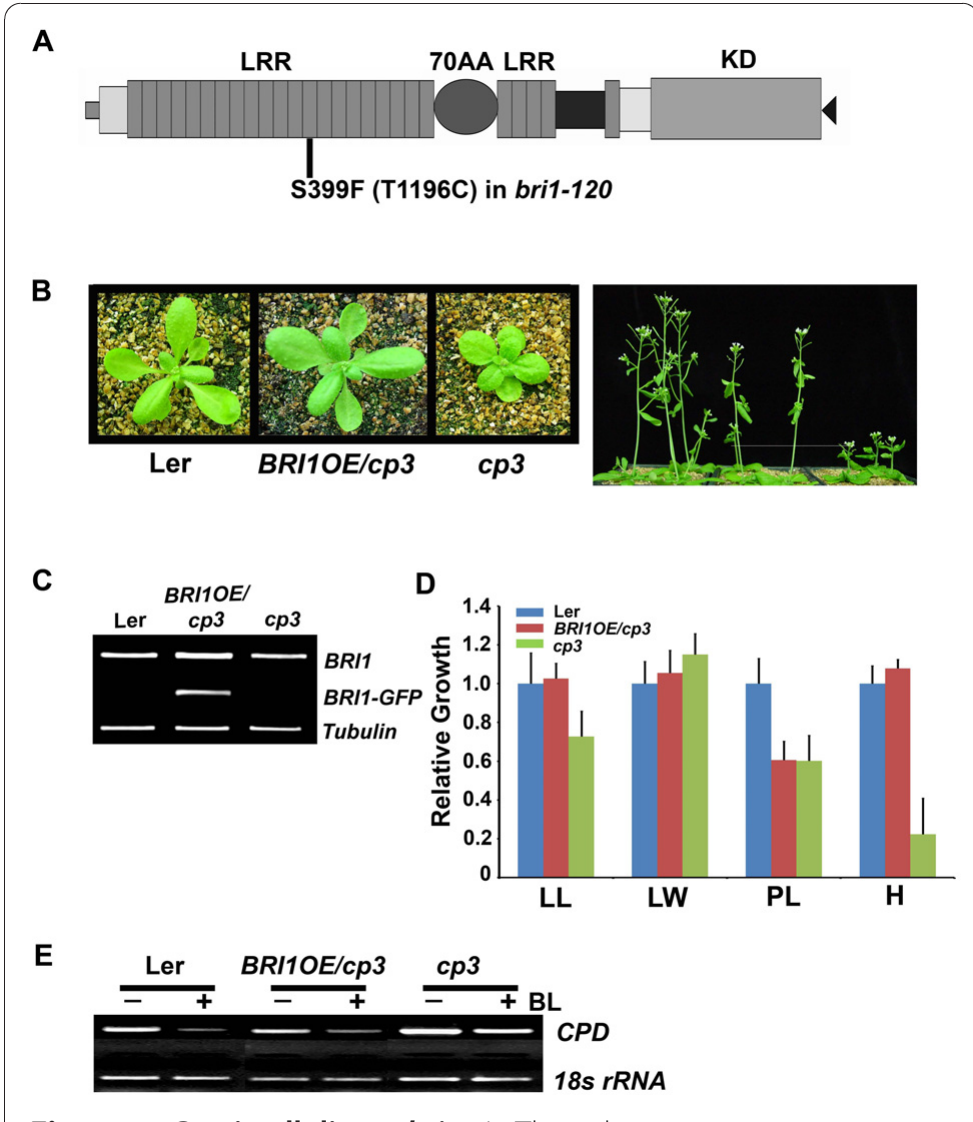
***Cp3 *is allelic to *bri1***. A. The schematic protein structure of BRI1 is shown and the mutation site of *bri1-120/cp3 *is marked in the 13^th ^LRR of the extracellular domain of BRI1. B. Overexpression of BRI1 rescued the *cp3 *mutant phenotypes. The pictures were taken of 3-week-old plants (left panel) and 5-week-old plants (right panel). C. RT-PCT analysis shown the expression of endogenous *BRI1 *and the *BRI1 *derived from the transgene in the *cp3 *mutant overexpressing *BRI1 *compared with those of Ler and un-transformed *cp3 *mutant. D. Quantitative growth criteria were measured from the transgenic *cp3 *overexpressing *BRI1*. LL: leaf length, LW: leaf width, PL: petiole length, and H: total height. Growth is represented as a relative value compared to that of Ler. Experiments were repeated twice. Error bars denote standard errors. E. Pattern of transcriptional inhibition of *CPD *expression in response to BL was restored in the transgenic *cp3 *overexpressing *BRI1*.

Based on the morphological phenotypes and reduced BL sensitivity of the *cp3 *mutant, we thought that *cp3 *may be one of the *bri1 *mutant alleles. Thus, we sequenced the genomic DNA spanning the *BRI1 *region in the *cp3 *mutant. We also sequenced the same region of Ler as a control. Since the Arabidopsis whole genome sequences are derived from the Col-0 ecotype, we found one mismatched nucleotide in the 3,512^th ^position from the open reading frame of the *BRI1 *sequence between Ler and Col-0. This nucleotide change causes an alteration from arginine to glycine in the 1171^st ^amino acid of BRI1. More importantly, we found an additional mismatched nucleotide at the 1196^th ^position from the open reading frame with a T to C change, resulting in a change from serine to phenylalanine at the 399^th ^amino acid position of BRI1 in the *cp3 *mutant (Figure 3A). The wild type Ler has a nucleotide T in the 1196^th ^position as in Col-0. Therefore, we reasoned that the nucleotide change at the 3512^th ^position of *BRI1 *in Ler is a natural polymorphism due to an ecotype difference between Ler and Col-0, and that the nucleotide change at the 1196^th ^position of BRI1 in the *cp3 *mutant compared to Ler causes its phenotypic changes.

To verify this notion, we generated a transgenic *cp3 *plant overexpressing *BRI1 *by introducing a *BRI1 *promoter-driven *BRI1:BRI1-GFP *construct. The growth of the *BRI1*-overexpressing *cp3 *plants was more similar to that of the wild type as compared to the non-transformed *cp3 *plants (Figure 3B). We confirmed that the *BRI1-GFP *transgene was highly expressed in the transgenic *cp3 *plants by RT-PCR analyses using primers that amplified transgene specifically (Figure 3C). The *cp3 *plants overexpressing *BRI1-GFP *showed nearly normal overall growth patterns with elongated leaves and petiole length as well as total height, similar to those observed with Ler (Figure 3D). In addition, the *cp3 *transgenic plants overexpressing *BRI1 *showed restored BL sensitivity, exhibiting a BL-induced transcriptional inhibition of *CPD *expression (Figure 3E). These results suggest that the growth retardation of the *cp3 *mutant accompanied by the dark green coloring is caused by a mutation in the extracellular domain of BRI1. Therefore, we renamed the *cp3 *mutant *bri1-120*, referring to the order of naming for *bri1 *mutant alleles [4]

### BRI1(S399F) protein is localized in plasma membrane and the overexpression of BRI1(S399F) in *bri1-301 *resulted in the leaf elongation of *bri1-301 *and co-suppression of the endogenous *bri1-301*

**Figure 4:**
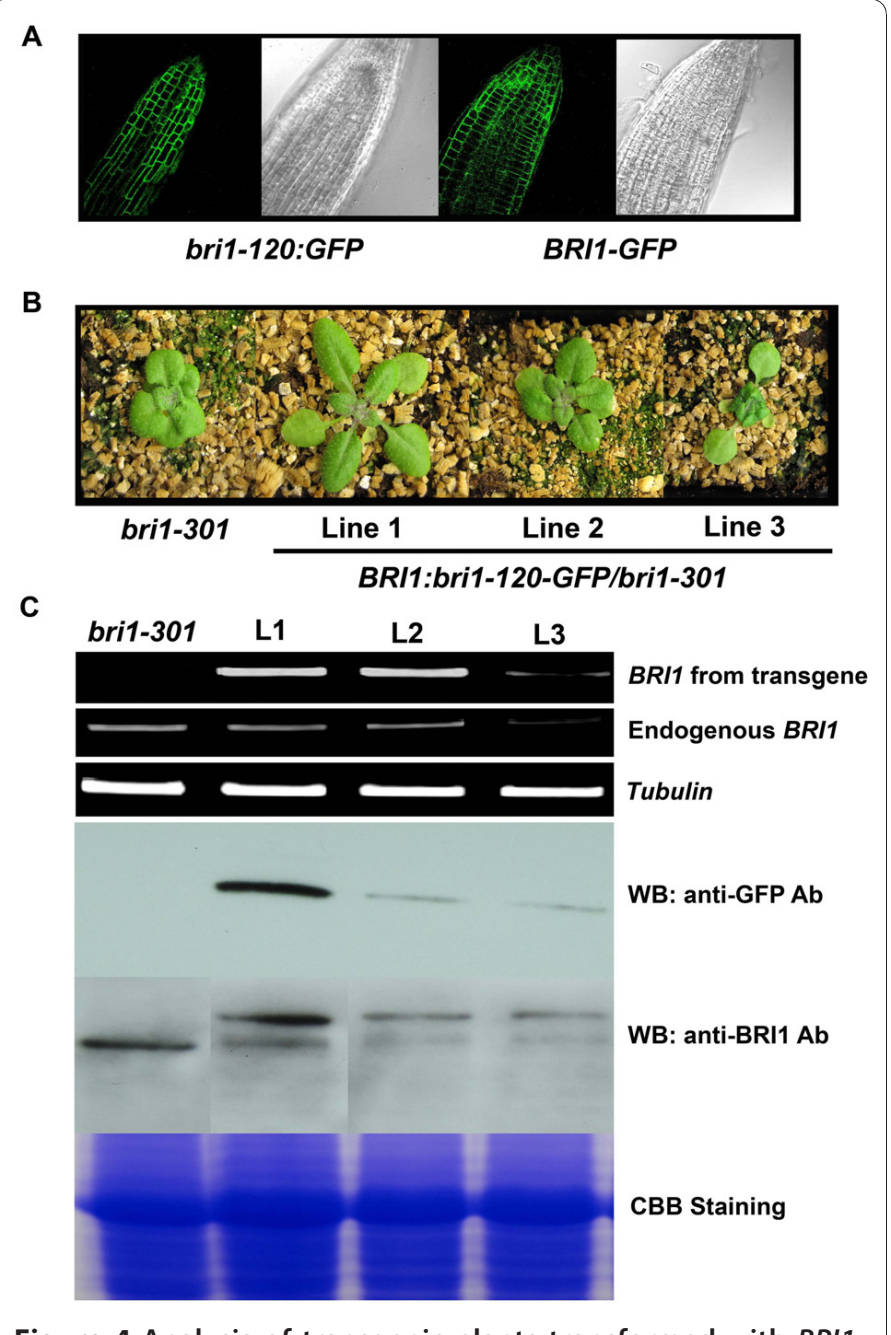
**Analysis of transgenic plants transformed with *BRI1:bri1-120-GFP***. A. Confocal microscopic observations of the GFP signal fused with BRI1(S399F) or intact BRI1 were performed on the root tips. Microscopic features of the same root tissues under bright-field were are shown side-by-side. B. Overexpression of *BRI1:bri1-120-GFP *in *bri1-301 *led to an allelic series of the *bri1 *phenotype. Representative fully rescued (Line 1), intermediate (Line 2), and strong *bri1 *mutant-looking transgenic *bri1-301 *plants are shown with un-transformed *bri1-301*. C. Analysis of *BRI1 *expression and determination of BRI1 protein amount in transgenic *bri1-301 *overexpressing *BRI1:bri1-120-GFP *detected by anti-GFP antibodies and anti-BRI1 antibodies.

We introduced nucleotide C instead of T at the 1196^th ^position of *BR1 *by site-directed mutagenesis to generate the *bri1-120 *mutated *BRI1*, using the *BRI1-GFP *construct as a template. The resulting construct (*BRI1:bri1-120-GFP*) was transformed into the wild type Col-0, *bri1-301 *plants to produce a mutated BRI1(S399F). After the wild type plant was transformed with *BRI1:bri1-120-GFP*, we first observed the intracellular localization of the BRI1(S399F) protein using a confocal microscope by detecting the GFP that was fused with BRI1(S399F) in the plasma membrane of the cells (Figure 4A), which indicated that *bri1-120 *possesses the plasma membrane-localized BL receptor, although BRI1(S399F) may not be fully functional protein. In comparison, the mutated BRI1 proteins, BRI1(C69Y) in *bri1-5 *and BRI1(S662F) in *bri1-9*, in which both mutations are in the extracellular domain of BRI1, are known to be localized to the endoplasmic reticulum (ER) [17].

We also produced transgenic *bri1-301 *overexpressing mutated BRI1(S399F) to examine whether the additional BRI1 proteins are able to rescue the *bri1-301 *mutant phenotypes or not, although the transgenic plants have two mutated forms of BRI1 derived from the *bri1-301 *and *bri1-120 *mutations, respectively. From this analysis, we found that all of the transgenic *bri1-301 *displayed phenotypes that were wild type in appearance, with less compact rosette leaves due to elongated petioles and leaves, even in the T1 generation. In subsequent generations, a phenotypic recovery of *bri1-301 *achieved by the overexpression of *BRI1:bri1-120-GFP *was observed in most of the plants. However, some plants showed only a partial recovery of the *bri1-301 *phenotype, and a few plants displayed stronger mutant phenotypes compared to the non-transformed *bri1-301 *(Figure 4B). And these phenotypic differences still remained in inflorescent adult stage (Additional file 2A). We attributed the phenotypic differences of the transgenic *bri1-301 *overexpressing *BRI1:bri1-120-GFP *to the co-suppression of *BRI1 *gene. The *BRI1 *transcripts derived from the endogenous *BRI1 *and the transgene were shown to be inversely correlated with phenotypic severity by RT-PCR analyses using primers that amplified each gene specifically (Figure 4C). Co-suppression was first shown in petunia, in which the transgene, chalcone-synthase A, caused transcript loss due to the degradation of the homologous endogenous gene [18]. Since then, it has been regarded as eukaryotic post-transcriptional gene silencing. We also performed a western blot analysis of the total proteins from the plants showing representative phenotypes using the anti-GFP antibodies and anti-BRI1 antibodies. As shown in Figure 4C in the bottom panel, all the transgenic plants produced mutated BRI1(S399F) protein fused to GFP detected by anti-GFP antibodies, although the protein expression level is higher in the transgenic *bri1-301 *plant that was wild type in appearance. When we used anti-BRI1 antibodies that can detect both endogenous and transgene-derived BRI1 proteins, the same plant contained more BRI1 proteins. In contrast, much less BRI1 proteins were detected in the strong *bri1 *mutant-looking *bri1-301 *transgenic plant compared to the untransformed *bri1-301*. We also detected *bri1 *mutant-looking phenotypic alterations that were due to co-suppression in the wild type plant overexpressing *BRI1:bri1-120-GFP *(Additional file 3).

### *Bri1-301 *and *bri1-120 *complemented each other to form a functional BRI1 receptor

**Figure 5:**
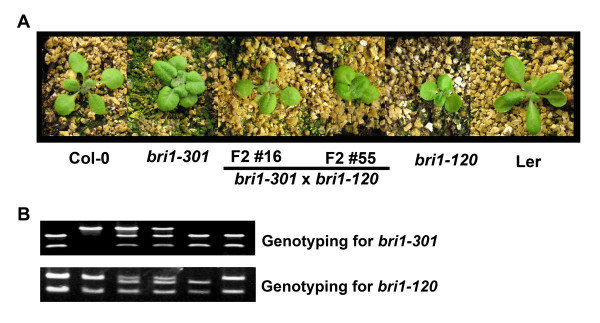
**Interallelic complementation between *bri1-120 *and *bri1-301***. Overall rosette phenotypes of the F2 plants produced by the genetic crosses of *bri1-120 *and *bri1-301*, whose genotypes were heterozygous for both mutations, are compared with parental mutant plants and wild type plants. Lower two panels show the confirmed genotype of the *bri1-120 *and *bri1-301 *mutation in each plant.

Based on the results above, we questioned whether an increased number of BRI1 proteins, although it is partially functional, is enough to mediate BR signaling with heterodimers consisting of mutated proteins, and if the heterodimerization between the BRI proteins containing an extracellular LRR domain mutation in *bri1-120 *and the BRI1 proteins with a cytoplasmic kinase domain mutation in *bri1-301*, reconstituted a fully functional BRI1 in the cells. To address these questions, we crossed *bri1-120 *with *bri1-301*. We expected that all of the F2 plants from this cross would exhibit semi-dwarf looking phenotypes, similar to both parental plants. However, when we analyzed 235 individual plants from the F2 generation, we found that the phenotypic segregation deviated slightly from the expected one. Thus, we grew and genotyped all of the plants using CAPS and dCAPS primers specific for the *bri1-301 *and *bri1-120 *mutations, respectively. More attention was directed toward the plants that were heterozygous both mutations in each homologous chromosome of the cell: the *bri1-301 *mutation residing on one homologous chromosome and the *bri1-120 *mutation on the other. Among these plants, approximately half showed compact rosettes and semi-dwarf statures similar to the parental mutant phenotypes, and the remaining half displayed rescued *bri1-301 *phenotypes in terms of overall rosette morphologies (Figure 5 and Additional file 2B). Because there are no additional BRI1 proteins added by the transgene in these crossed plants, it is suggested that the rescued *bri1-301 *phenotype resulted from the interallelic complementation that occurred between the *bri1-120 *and *bri1-301 *mutated alleles.

### Different BL sensitivity was observed in the *bri1-301 *transformed with *BRI1:bri1-120-GFP *and the *bri1-301 *crossed with *bri1-120*

**Figure 6:**
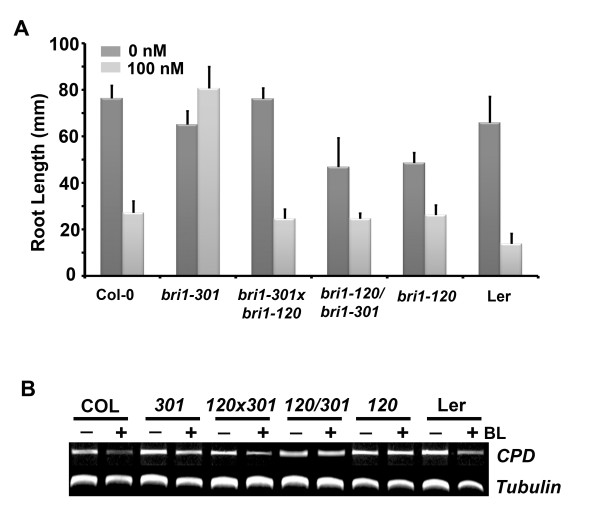
**BR sensitivity of the rescued *bri1-301 *plants**. A. Root growth was measured for the indicated plants grown vertically in 1/2 MS media or 1/2 MS media containing 100 nM of BL. Experiments were repeated three times. Error bars denote standard errors. B. Transcriptional inhibition of *CPD *expression in response to BL was determined in the rescued *bri1-301 *plants compared with that of control plants.

The results above indicate that both the overexpression of *bri1-120 *by transformation and the reconstitution of functional BRI1 by crossing it with *bri1-120 *restored the mutant phenotype of *bri1-301*. We also wanted to know whether BR sensitivity returns to normal in these plants. We examined root growth inhibition in the transgenic *bri1-301 *transformed with *BRI1:bri1-120-GFP *in the presence or absence of BL. Compared with the un-transformed *bri1-301 *and *bri1-120 *control plants, the root length of the transgenic *bri1-301 *plant in the absence of BL was shorter than that of non-transformed *bri1-301*, similar to that of *bri1-120*. Moreover, the root growth inhibition pattern exhibited after the BL treatment of the transgenic *bri1-301 *plant was similar to that of *bri1-120 *(Figure 6A). The rescue of the transcriptional inhibition of *CPD *expression in the transgenic *bri1-301 *by the overexpression of *BRI1:bri1-120-GFP *was not as dramatic as that observed in the wild type, either (Figure 6B). In comparison, the root lengths of the wild type-looking F2 plants crossed with *bri1-301 *and *bri1-120 *were more similar to the root length of the wild type. Also, the degree of inhibition of root growth showed similar patterns compared to the wild type (Figure 6A). The transcript level of *CPD *was reduced in response to BL to the same degree as seen in the wild type (Figure 6B). Taken together, these results suggest that the F2 plants crossed with *bri1-301 *and *bri1-120 *were similar to the wild type plant not only morphologically but also in terms of their cellular responsiveness to BL, leading to the strong assumption that these F2 plants contain a functional BRI1 (Figure 6A).These results suggest that the elongated rosette phenotype that has been frequently considered to be the BR sensitivity gauge may not be coupled with other assessment of BR sensitivity, such as root growth inhibition or *CPD *expression in response to BL.

## Discussions

### *BRI1-120 *revealed the importance of the LRR region in the extracellular domain of BRI1

The degree of phenotypic alteration caused by each *bri1 *allele depends on the specific affected mutation sites [4]. Mutants that have amino acid changes in the cytoplasmic kinase domain usually show very strong mutant phenotypes, which can be attributed to loss of BRI1 kinase activity. *Bri1-301 *is an exceptional case. Although *bri1-301 *was shown to be a kinase-inactive protein, the mutant plant exhibits only mild phenotypic changes. *Bri1-301 *contains two nucleotide changes (GG to AC) in the cytoplasmic kinase domain of BRI1, resulting in a change from Gly989 to Ile [19]. However, Gly989 is not a conserved amino acid, and its position is slightly out of the critical region of the kinase domain. So, it is possible that Gly989 is important for maintaining the proper conformation of the BRI1 protein to retain its kinase activity, but, not for controlling the kinase activity itself.

In comparison, most of the mutations in the extracellular domain of BRI1 produced relatively mild mutant phenotypes. A more thorough examination of the extracellular domain of BRI1 revealed that the 70-amino acid island domain and the subsequent four LRRs before the transmembrane domain are frequent mutation sites, indicating their functional importance to the BRI1 protein. In addition, the first cysteine pair before the beginning of the LRRs is thought to be critical for BRI1 as seen in the mutant *bri1-5 *(C69Y). So far *bri1-4 *is the only mutant in which the mutation occurred in the LRR regions preceding the 70-amino acid island domain [16]. However, a 10-bp deletion in the 3^rd ^LRR of BRI1 in *bri1-4 *introduced a premature stop in translation and did not provide any clues regarding the functional importance of the LRR domains of BRI1.

In this study, we analyzed the BR-related phenotypes of *cp3 *grown from the CS48 seeds obtained from ABRC to have more natural mutants with similar morphologies to known *bri1 *mutants, although the phenotypic strength of *bri1-120 *is relatively weak compared to other *bri1 *mutants, such as *bri1-5 *or *bri1-9*. *Cp3 *has the *COMPACTA3 *(*cp3*) mutation, and *cp3 *mutants show altered phytochrome A signaling [20]. However, the mutated gene has not been characterized yet. From the direct sequencing of the genomic DNA region containing *BRI1*, we found that this plant contains a mutation in *BRI1 *called *bri1-120*. Bri1-120 contains phenylalanine instead of serine at the 399^th ^position in the 13^th ^LRR due to a nucleotide change (T to C) at the 1196^th ^position (Figure 3A). When we overexpressed wild type BRI1 in *bri1-120*, mutant phenotypes of *bri1-120 *were rescued not only morphologically but also in terms of their sensitivities to BR (Figure 3). Overexpression of the bri1-120 protein in wild type plants produced transgenic plants with *bri1 *mutant phenotypes (Figure 4 and Supplementary Figure 2). We believe that *bri1-120 *is the first example of a natural mutant allele with a point mutation in the LRR region of the extracellular domain of BRI1. These results suggest that the LRR region before the 70-amino acid island domain is also important in maintaining a fully functional BRI1.

Tandem array of repeating LRR are known to provide protein-protein interaction motif [21]. The plant-specific LRR motif out of seven subfamilies contains 23-25 amino acids that form an extended β-strand connected with an α-helix by a loop [22]. Especially, first 11 amino acid residues (LxxLxLxxNxL) in LRR are highly conserved and corresponds the region forming β-strand and loop [21,23]. Leucine residues can be compatible with isoleucine (I), valine (V), and phenylalanine (F), which form the hydrophobic core [24]. Asparagine (N) in the 9^th ^position is important for half-turn in LRR unit, and serine or threonine are the preferred amino acid in the 8^th ^position, just before the asparagine [25]. We found that the first part of amino acid sequence in the 13^th ^LRR of BRI1 (LLTLDLSSNNF from 392^nd ^to 402^nd ^amino acid in BRI1) is well matched with the known consensus sequence. Compared with that, the serine residue at the 399^th ^position of BRI1 in front of the asparagines is changed to phenylalanine in *bri1-120 *mutant. Regarding that serine or threonine is able to form an additional hydrogen bond with other part of proteins, it is highly possible that hydrophobic phenylalanine instead of serine residue in *bri1-120 *causes conformational change of LRR motif in the BRI1. Among other genes encoding the LRR-RLKs, *CLAVATA1 *(*CLV1*) which involves in meristem differentiation has been reported to have three missense mutant alleles within LRRs: *cla1-10 *in LRR4, *clv1-4 *in LRR5, and *clv1-8 *in LRR9. These mutations were likely to be harmful for the dimerization of CLV1 with other receptors [26]. The HAR receptor that regulates the nodulation in legumes possesses 21 LRRs. Mutation in the LRR7 in *har1-4*, which alters β-strand structure, led to the reduced ligand binding [27]. Therefore, it is possible that conformational changes due to a mutation in the 13^th ^LRRs of BRI1 affect receptor dimerization or reduce ligand binding capacity. Recently, several mutants generated by the TILLING method were reported to have amino acid changes in the LRR region of the extracellular domain of BRI1 [28] (http://tilling.fhcrc.org), and they are awaiting further analysis to reveal the functional significance of the LRR domain of BRI1.

### Interallelic complemented *bri1-301 *showed different BL sensitivity as compared to the *bri1-301 *overexpressing a *BRI1:bri1-120-GFP*

There have been many reports that the compact and downward-curling rosette leaves that are considered to be weak *bri1 *mutant phenotypes can be restored by the overexpression of the genes encoding the positive regulators of BR signaling, such as BAK1 [5,6], BSK1 [29] and BES1 [10], and BRI1 itself [8]. *Bri1-9*, *bri1-5 *and *bri1-301 *are frequently used in these types of studies. Here, we showed that the phenotypic defects in the rosette leaves of *bri1-301 *can be restored in two ways. First, we overexpressed *BRI1:bri1-120-GFP*, causing the *bri1-120 *mutation in *bri1-301*, and we showed that the transgenic *bri1-301 *displayed an elongated leaf and petiole growth pattern similar to that of the wild type (Figure 4B and 4C). Secondly, we generated plants by crossing *bri1-120 *with *bri1-301*. Receptors that require the assembly of homodimers in order to become active signaling complexes were interallelically complemented [30,31]. However, to date, it has not been elucidated that whether the *bri1 *alleles that have the extracellular domain mutation are able to complement kinase-inactive *bri1 *alleles. By showing that more than half of the F2 plants had perfectly wild type-looking overall rosette morphologies, we demonstrated a successful interallelic complementation with two different *bri1 *alleles (Figure 5). The possibility that the genetic recombination between one homologous chromosome with a *bri1-120 *mutation and the other homologous chromosome with a *bri1-301 *mutation occurs during the self fertilization of a F1 progeny after the initial cross, resulting in a homologous chromosome without either mutation, cannot be completely ruled out. However, that event seems to occur very rarely, because both mutations are less than 2 Kb apart.

Interestingly, during our analysis, we found significant differences in growth patterns and the BR sensitivities between the *bri1-301 *plants rescued by the genetic cross with *bri1-120 *and the *bri1-301 *plants rescued by the transformation of a *BRI1:bri1-120-GFP *construct. The overall rosette phenotype of the rescued *bri1-301 *plants generated by any one of the methods was similar to that of the wild type plants. However, the *bri1-301 *plants overexpressing BRI1(S399F) due to the transformation of *BRI1:bri1-120-GFP *showed reduced root and hypocotyl growth in normal growth conditions compared to the wild type plants. Moreover, the BR sensitivities of these plants were similar to the BR sensitivity of *bri1-120 *based on the inhibition of root growth and *CPD *expression in response to BL. On the other hand, both root and hypocotyl growth and BR sensitivity almost completely reverted to wild type levels in the plants heterozygous for each mutated allele due to the cross of *bri1-301 *and *bri1-120 *(Figure 6). It is possible that although the bri1-301 phenotypes could be rescued by both a transgenic approach, transformation of *BRI1:bri1-120-GFP *gene, and a genetic cross with bri1-120, different growth pattern in detail and the BR sensitivity between both lines were resulted from the more accumulation of the BRI1-120-GFP proteins in transgenic *bri1-301*, because expression level of transgene was diverse in each transgenic plant. We also cannot rule out the possibility that the increased amount of BRI1-120-GFP proteins in transgenic *bri1-301 *affected only rosette development with unknown mechanisms yet. Taken together, these results suggest that observing the shape of the rescued rosette, including the elongated leaves and petioles, is not likely to be a precise way to determine BR sensitivity. A recent publication supported this view. Albrecht *et al. *(2008) [32] reported that the overexpression of *AtSERK4 *in *bri1-301 *led to the appearance of the rescued compact rosette leaves but did not promote hypocotyl growth. Additionally, we previously showed similar phenomena when *BAK1 *was overexpressed in *bri1-301 *[33]. Conventionally, several indicators, such as the conversion of the rosette leaf phenotypes from compact, curled and dark-green elongated, the inhibition and promotion of the root and hypocotyl growth, respectively, the transcriptional inhibition of *CPD *expression, and the BL-induced accumulation of dephosphorylated BES1, have been used to denote normal BR sensitivity. We believe that each experimental method represents a different degree of BR sensitivity. In that sense, the rescued rosette phenotype does not reflect heightened BL sensitivity as compared to any other method. However, the changes observed in the outward appearance of the weak *bri1 *mutant phenotype can still be regarded as useful indicators the genetic suppressor screening of *bri1 *mutants to find additional regulators involved in BR signaling. A BRI1 co-receptor BAK1 [6], BRS1 (a secreted carboxpeptidase) [34], BRL1 (BRI1-like1) [35], BSU1 (a serine/threonine protein phosphatase) [36], and BEN1 (a dihydroflavonol 4-reductase-like protein) [37], and recently published TCP1 (a transcriptional modulator of *DWARF4*, BR biosynthetic gene) [38] are examples of *bri1 *suppressors identified in the activation-tagged *bri1-5*. In addition, the proteins involved in ER quality control were revealed allele-specifically in the genetic suppressor screening of EMS-mutagenized *bri1-9 *[39-41]. *Bri1-301 *was also used for the suppressor screening in the activation tagged pools, resulting in the identification of several ATBS genes, including one encoding a bHLH transcription factor that regulates BR signaling (ATBS1) [42] and YUCCA, which is involved in tryptophan-dependent auxin biosynthesis (ATBS3 to ATBS6) [43]. These results imply that the suppressor screening of *bri1 *mutant alleles with rosette leaf phenotypes can allow for the mining of genes related to diverse cellular functions in addition to BR signaling. We believe that *bri1-120 *is a suitable mutant allele for this purpose. We are currently performing genetic screening to search for modulators of *bri1-120*, to expand the understanding of the functions of this gene.

## Conclusions

In summary we demonstrated that the mutant previously referred to as *cp3 *that shows retarded growth and reduced BR sensitivity is allelic to *bri1*, and we renamed it *bri1-120*. The analysis of a point mutation in the 13^th ^LRR that resides before the 70-amino acid island portion of the extracellular domain of BRI1 has indicated that this specific LRR region is critical for proper BRI1 functioning. Using *bri1-120 *and *bri1-301*, we revealed that interallelic complementation is able to occur between the extracellular domain mutant allele and the cytoplasmic kinase-inactive mutant allele of *BRI1 in vivo.*

## Methods

### Plant growth condition

We used *Arabidopsis thaliana *Landsberg (Ler) as the wild type for the comparison with phenotypic changes of *bri1-120 *(seeds from CS48) and used *Arabidopsis thaliana *Columbia (Col-0) as the wild type for the comparison with phenotypes of the transgenic *bri1-301 *plants. All transgenic plants used here were made by floral dipping into suspensions of *Agrobacterium tumerfaciens *(GV3101) containing appropriate binary plasmid constructs. Seed sterilization was performed by washing the seeds with 75% ethanol containing 0.05% Tween-20 for 15 minutes, and then washing them twice with 95% ethanol. Sterilized seeds were plated in 1/2 MS (Duchefa) containing 0.8% phytoagar. After stratification at 4°C for 2 days, plates were transferred to a growth room set at 22°C under long-day conditions (16 hours L/8 hours D). To observe the plant phenotypes, the seeds were sown directly onto soil (Sunshine #5) top-layered with fine particles of vermiculite.

### Construction of plasmids

The plasmid containing the *bri1-120 *mutation in *BRI1 *to express the mutated BRI1 protein, BRI1(S399F), was made by in vitro site-directed mutagenesis using a QuickChange Site-Directed Mutagenesis Kit (Stratagene) with *pPZP212-BRI1:BRI1-GFP *as a template. The sequences of the primers used were a 5-cgttagatctcagcttcaacaatttctccgg-3' (forward) and 5'-ccggagaaattgttgaagctgagatctaacg-3' (reverse). All of the resulting plasmids were fully sequenced to confirm the presence of the intended changes and the absence of other alterations. After confirmation, the plasmid, *BRI1:bri1-120-GFP*, was transformed into wild type and *bri1-301 *plants by *Agrobacterium tumefaciens*-mediated floral dipping.

### Confocal microscopic analysis of the subcellular localization of BRI1(S399F)

The localization pattern of BRI1(S399F) was analyzed by examining the root tips of 5-day-old *BRI1:bri1-120-GFP *transgenic seedlings using a Zeiss LSM510 Meta confocal microscope with excitation set at 488 nm and a 500-530-nm band-path filter was used to detect the GFP.

### Root growth inhibition assay

To determine the BR sensitivity of the plants, the sterilized seeds of interest were placed in a line on 1/2 MS containing 0.8% phytoagar plates supplemented with or without brassinolide (BL) at the indicated concentrations. The seeds of the different plants of interest were seeded in the same plate to minimize ambient differences. Three sets of plates were plated vertically and grown for 10 days at 22°C under long-light conditions (16 hours L/8 hours D) for root elongation. Root lengths were measured for 20-30 seedlings in each line. To determine the hormone sensitivity of *bri1-120*, we added 20 μM of IAA, GA, kinetin, and ACC and 50 μM of JA to 1/2 MS MS plates and processed them the same way. All of the chemicals were purchased from Duchefa Biochemie except IAA (Sigma Aldrich) and BL (Synthchem. Inc.) All experiments were repeated twice.

### *CPD *expression analysis

We grew the sterilized seeds of interest on the 1/2 MS (Duchefa) containing 0.8% phytoagar plates supplemented with or without brassinolide (BL) for 10 days and extracted total RNA from each seedling. For the northern hybridization, the total RNA was run on a formaldehyde-containing 1% agarose gel, blotted onto a nylon membrane (GE Healthcare) and hybridized with the ^32^P-labeled CPD probe (^32^α-P-dCTP, 10 mCi/mol, IZOTOP) at 42°C in a hybridization solution (1M NaCl, 1% SDS, 1% dextran sulfate (Sigma Aldrich), and 50% formamide). For the RT-PCR analysis, the RNA was treated with RNase-free RQ1 DNases (Promega), and the first-strand cDNA was synthesized using the SuperscriptⅢ-MMLV reverse transcriptase (Invitrogen) and oligo d(T_15_) primer. The same aliquot of first-strand cDNA was used as a template in the second polymerase chain reaction, in which the *CPD *transcript was amplified for 23 cycles with the primers CPD-RTF: 5'-gccttcaccgcttttctcctcctc-3' and CPD-RTR: 5'-atttgacggcgagagtcatgatcg-3'.

### Confirmation of BRI1 expression by RT-PCR analysis

RNAs were purified from the seedlings grown for two weeks on 1/2 MS plate, and treated with RNase-free RQ1 DNase (Promega). First-strand cDNA synthesis was performed using the SuperscriptⅢ-MMLV reverse transcriptase (Invitrogen) according to manufacturer's protocol. Second step of polymerase chain reactions were performed with the same aliquot of first-strand cDNA as a template. Polymerase chain reaction was as followings: pre-denaturation at 94°C for 4 min., denaturation at 94°C for 30sec., primer-annealing at 52°C for 30 sec., elongation at 72°C for 30 sec. for 22 cycles, and post-elongation at 72°C for 7 min. The primer sequences for detection of endogenous *BRI1 *expression are 15F7: 5'-tgcgatggatacgcatttaa-3' (forward) and BRI1 3'UTR: 5'-tcggactgacccttagatg-3' (reverse). The primer sequences for detection of transgene-derived *BRI1 *expression are GFPSEQF: 5'-acaacatcgaagacggcggcgtg-3' (forward) and KH002: 5'-cagtaggattgtggtgtgtgcgc-3' (reverse). The expression of each gene was normalized to β-*Tubulin *with primers of TUBF 5'-atgcgtgagattcttcacatcc-3' (forward) and TUBR 5'-tgggtactcttcacggatcttag-3' (reverse).

### Genotyping of *bri1-120 *and *bri1-301 *mutations

For the *bri1-301 *genotyping, the genomic DNA region adjescent to the *bri1-301 *mutation was amplified in a polymerase chain reaction (PCR) with the primer set 5'-ggaaaccattgggaagatca-3' (forward) and 5'-gctgtttcacccatccaa-3' (reverse) and then digested with *DPN*Ⅱ. One of the restriction sites for *DPN*Ⅱ in the PCR-amplified fragment is lost in *bri1-301*, so DNA fragments with different sizes can be distinguished in the 1% agarose gels after electrophoresis. For the *bri1-120 *genotyping, we PCR-amplified the genomic DNA with specifically designed dCAPS primers 5'- ccgcttcgttgctaacgttagatctaagct-3' (forward) and 5'-ccagttaagattggtacagttacttaaacc-3' (reverse), to generate a *Hind*Ⅲ site only in *bri1-120*. *Hind*Ⅲ-digested PCR products were run on a 3% agarose electrophoresis gel. Wild type Col, Ler, *bri1-120*, *bri1-301*, and the F1 plants crossed with *bri1-120 *and *bri1-301 *were always included in the experiments as controls.

### Detection of BRI1 proteins by western blot analysis

Total protein crude extracts were prepared from 3-4 leaves of 3-week-old soil-grown plants with the extraction buffer (50 mM HEPES (pH 7.4), 10 mM EDTA, 0.1% Triton X-100, and a protease inhibitor cocktail (1 tablet/50 mL, Roche)). Equal amounts of total protein were separated by 7.5% SDS-PAGE and blotted onto a PVDF membrane (Bio-Rad) with the BIO-RAD Mini PROTEAN and Criterion systems, respectively. A western blot analysis was carried out with anti-BRI1 antibodies and peroxidase-conjugated secondary antibodies (Goat anti-rabbit IgG, Pierce). Protein bands were visualized with an ECL plus western blotting detection system (GE Healthcare).

## Authors' contributions

**YS **designed and performed all of the experiments. **MML **participated in designing the experiment involving the genetic crosses of *bri1-120 *and *bri1-301*. **JL **provided the *bri1 *mutant seeds and helped with manuscript preparation. **KHN **is the primary investigator for this study; she conceived and coordinated the whole study, and wrote and revised the manuscript. All authors read and approved the final manuscript.

## Supplementary Material

Additional file 1 **Test for BR sensitivity of *cp3***. *Cp3 *and Ler were grown on 1/2 MS for 9 days, and then 1 μM of BL and mock treatment were applied to the plates. Photos were taken after overnight incubation. Click here for file

Additional file 2 **Plant Phenotypes of inflorescence stage**. A. Three representative transgenic *bri1-301 *plants overexpressing of *BRI1:bri1-120-GFP *shown in figure 4B were taken pictures after 7 weeks' growth. B. Adult stage phenotypes of F2 plants produced by the genetic crosses of *bri1-120 *and *bri1-301 *shown in figure 5A are exhibited with a *bri1-120 *single mutant. Click here for file

Additional file 3 **Overexpression of *BRI1:bri1-120-GFP *in wild type**. A. Transgenic plants that show no discernible phenotypic changes (Line1) or display strong *bri1 *mutant-looking phenotypes (Line 2) are shown with an un-transformed wild type plant. B. Analysis of *BRI1 *expression from the phenotypically representative transgenic plants. Click here for file
