# Supplementary figures and images for: Characterization of cp3 reveals a new bri1 allele, bri1-120, and the importance of the LRR domain of BRI1 mediating BR signaling
Source: BMC Plant Biol. 2011 Jan 11;11:8. doi: 10.1186/1471-2229-11-8 (PMC3024917; doi:10.1186/1471-2229-11-8)

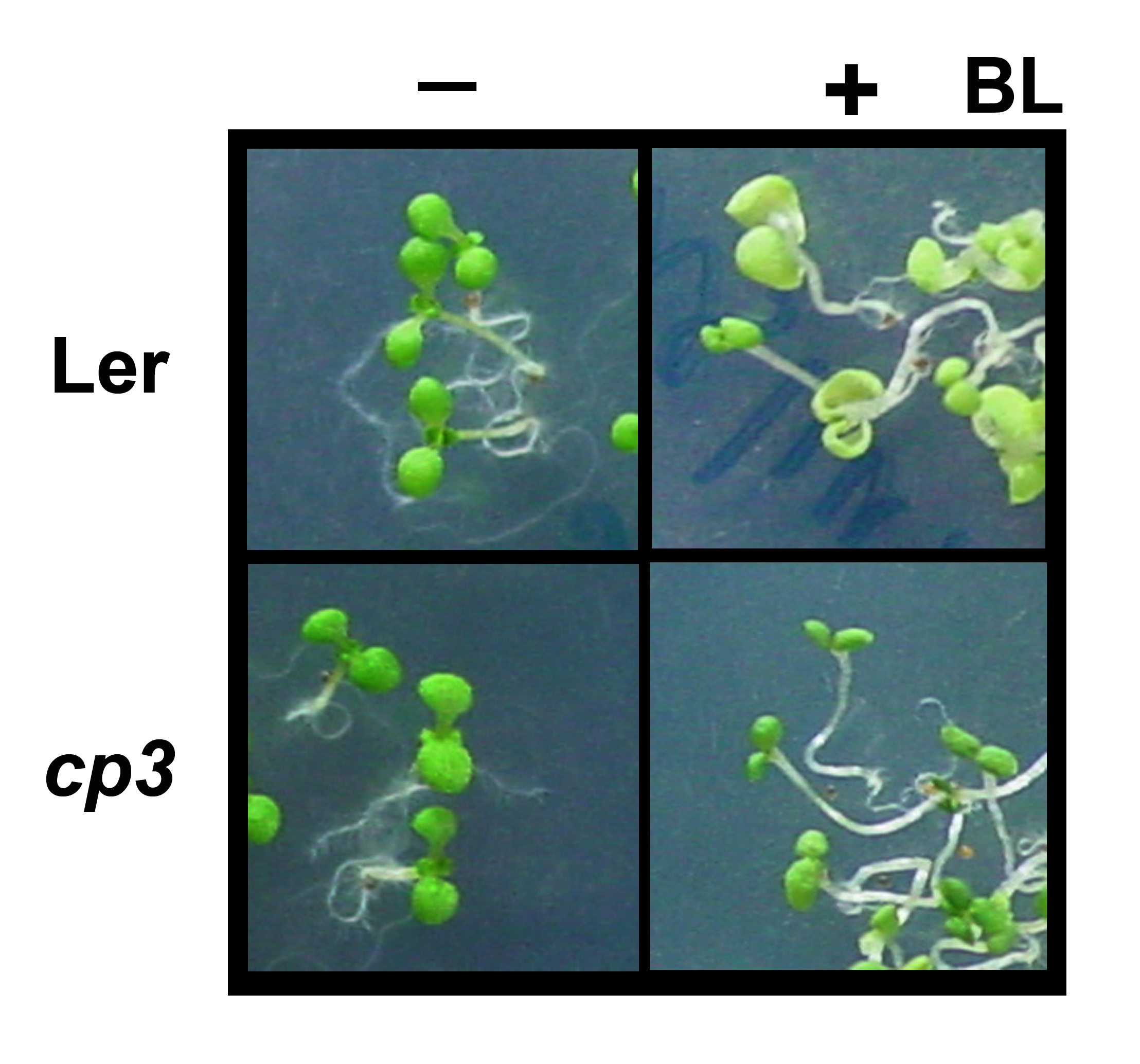

Supplement: Additional file 1 — Test for BR sensitivity of cp3. Cp3 and Ler were grown on 1/2 MS for 9 days, and then 1 μM of BL and mock treatment were applied to the plates. Photos were taken after overnight incubation. [file 1471-2229-11-8-S1.TIFF]

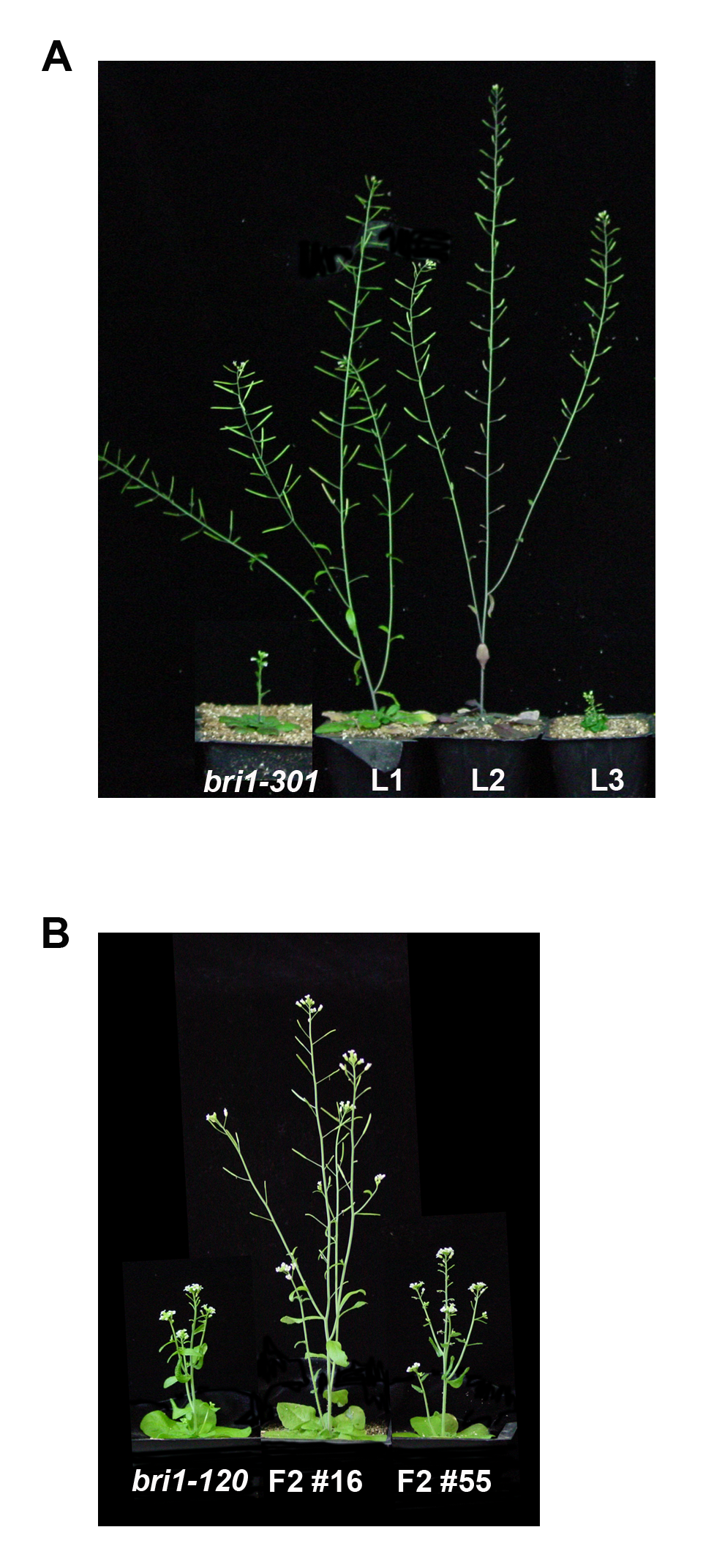

Supplement: Additional file 2 — Plant Phenotypes of inflorescence stage. A. Three representative transgenic bri1-301 plants overexpressing of BRI1:bri1-120-GFP shown in figure 4B were taken pictures after 7 weeks' growth. B. Adult stage phenotypes of F2 plants produced by the genetic crosses of bri1-120 and bri1-301 shown in figure 5A are exhibited with a bri1-120 single mutant. [file 1471-2229-11-8-S2.TIFF]

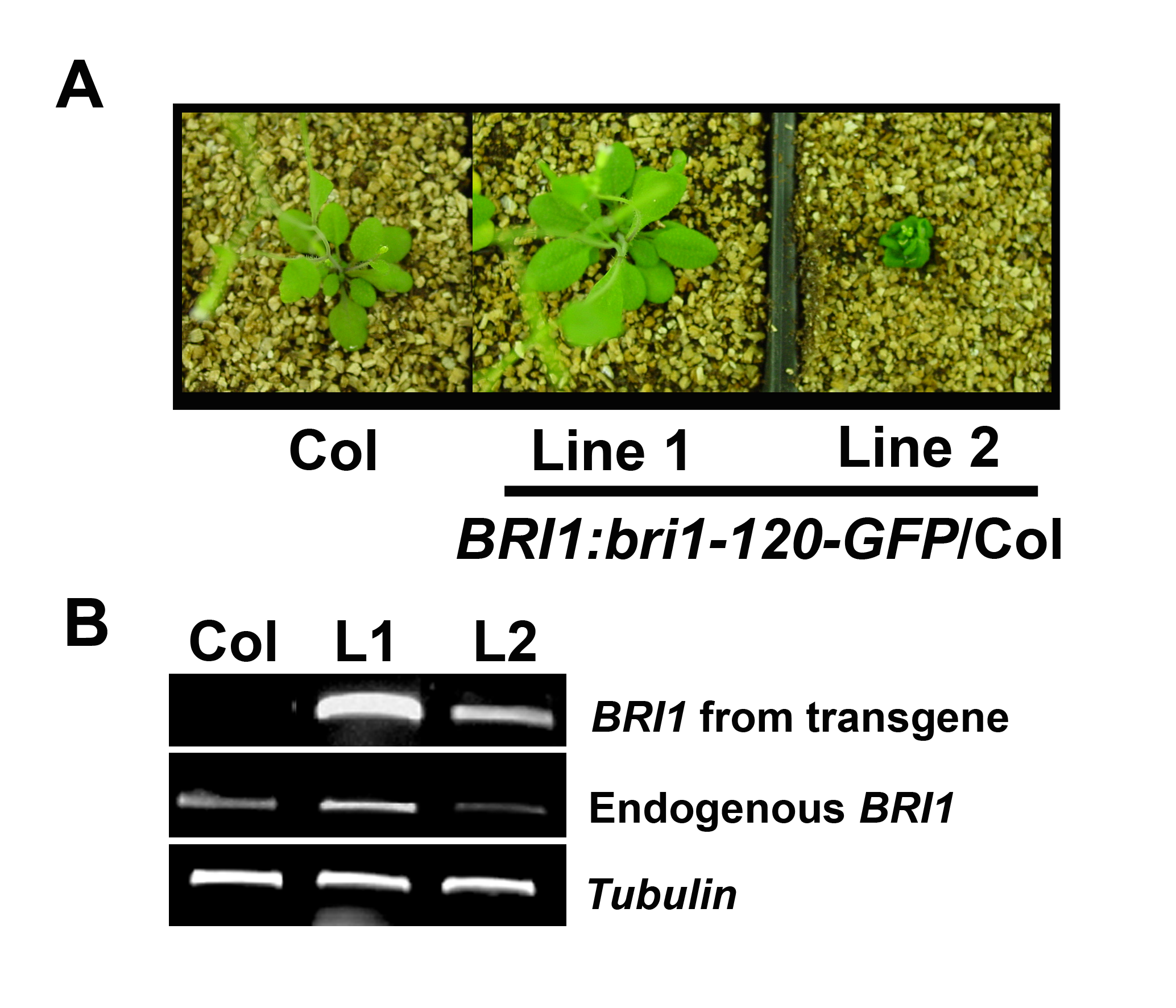

Supplement: Additional file 3 — Overexpression of BRI1:bri1-120-GFP in wild type. A. Transgenic plants that show no discernible phenotypic changes (Line1) or display strong bri1 mutant-looking phenotypes (Line 2) are shown with an un-transformed wild type plant. B. Analysis of BRI1 expression from the phenotypically representative transgenic plants. [file 1471-2229-11-8-S3.TIFF]
